# Maxillary Incisors of the Horse before and at the Beginning of the Teeth Shedding: Radiographic and CT Study

**DOI:** 10.3390/ani10091618

**Published:** 2020-09-10

**Authors:** Francisco Miró, Carla Manso, Andrés Diz, Manuel Novales

**Affiliations:** 1 Department Comparative Anatomy and Pathology, University of Córdoba, Ctra. de Madrid, 14071 Córdoba Ctra, Spain; an1dipla@uco.es; 2 Veterinaries Specialist in Equine dentistry, Pedro Laín Entralgo 8, Boadilla del Monte, 28660 Madrid, Spain; cpmanso@hotmail.com; 3 Diagnostic Imaging Service, Veterinary Teaching Hospital, University of Córdoba, Ctra. de Madrid, 14071 Córdoba, Spain; pv1nodum@uco.es

**Keywords:** computed tomography, development, incisor, radiograph

## Abstract

**Simple Summary:**

Although much is known about equine dentistry, there is a period of the horse’s life, prior to teeth shedding, in which there is lack of knowledge related to the development of deciduous incisors and dental germs of permanent incisors. To gain insight into the radiographic appearance of maxillary deciduous incisors and dental germs of maxillary permanent incisors during this period, a radiographic and computed tomography study of 25 horse skulls was made. Data regarding morphology and development were obtained. The results of the present study indicate that radiographic intraoral images are suitable to identify the grade of development of the dental germs of permanent incisors in horses before dental change. A detailed description of the radiographic appearance of deciduous incisors and dental germs of permanent incisors will help clinicians to expand their knowledge for diagnostic and treatment purposes.

**Abstract:**

To gain insight into the radiographic appearance of maxillary deciduous incisors and dental germs of maxillary permanent incisors in the period prior to teeth shedding, radiographs and computed tomography (CT) of 25 horse skulls, with an estimated age of between 12 and 42 months, were studied. Data regarding morphology and development were obtained. Dental germs of first maxillary permanent incisors were identified radiographically as rounded radiolucent areas at the level of the apical parts of the first deciduous incisors, in skulls with an estimated age of twelve months. The first sign of crown mineralization of these dental germs appeared in skulls supposedly a few months older. Before teeth shedding, the unerupted, mineralized crowns of the first permanent incisor could be identified radiographically relatively caudal to the corresponding first deciduous incisors. The results of the present study indicate that radiographic intraoral images are suitable to identify the grade of development of the dental germs of maxillary permanent incisors. A detailed description of the radiographic appearance of deciduous incisors and dental germs of permanent incisors will help clinicians to expand their knowledge for diagnostic or treatment purposes.

## 1. Introduction

In horses, the grade of development of deciduous and permanent teeth, including incisors, has always been of great interest for veterinary clinicians to determine their age. Besides, knowledge of dental and periodontal regions is needed when dental and periodontal disorders, such as malocclusions, dental fractures, persistent deciduous teeth, supernumerary teeth, traumas, etc., are present in young animals [1,2]. Although visual examination of the mouth and radiography have always been the most-used methods by veterinary clinicians [3], recent diagnostic imaging procedures, such as computed tomography (CT), provide complementary and more precise information on dental examination in horses [4,5,6,7,8,9]. 

Some authors have reviewed and summarized the current knowledge of equine dental and periodontal anatomy [10,11,12,13] and the three-dimensional appearance of teeth, as well as their individual composition of dental hard structures [14]. Throughout the life of a horse, specific changes occur to the appearance of its teeth, and dental examination therefore provides the most convenient mean for age determination and diagnostic purposes. In this sense, there is information relating morphological characteristics of deciduous and permanent incisors observed by visual examination with the age of the horse [11,15,16,17]. Equine teeth, pertaining to the high crowned hypsodont teeth, are subjected to continuous dental wear [18], and the process of change from deciduous to permanent dentition involves a complex mechanism of development of the dental germs. Time of radiographic appearance and grade of development of dental germs are well-known for deciduous and permanent cheek teeth [19,20], but very few data are available for incisor teeth [21,22]. The latest studies focus on the disorders involving the incisive bone, maxillary incisors, and periodontal structures and show very few radiographic data of dental germs of the permanent incisors. These few data and the clinicians’ experience indicate that there is a period of the horse’s life in which the deciduous incisors and the dental germs of permanent incisors undergo different stages that could be identified radiographically. The referred period would extend from the complete eruption of deciduous incisors, some months before twelve months of age, to the eruption of the first permanent incisor, at the age of thirty months [11,15,23]. High quality intraoral dorsoventral radiographs are an excellent tool to study the maxillary incisors [1,23]. Moreover, modern imaging techniques such as computed tomography complement and overcome the limitations of two-dimensional radiographic images. To accurately diagnose and formulate a treatment plan, evaluation of dental disorders requires a complete maxillofacial–oral examination and supplemental imaging means [21]. Some authors have compared and validated the accuracy of CT and radiographic imaging in detecting cheek teeth disorders in horses [24]. As stated above, in horses, there is a lack of radiographic data on deciduous incisors and dental germs of permanent incisors in the period before and at the beginning of the teeth shedding. Hence, a detailed description of the radiographic appearance of the referred structures in the referred period will help clinicians to expand their knowledge for diagnostic or treatment purposes.

We hypothesized that, in young horses before shedding, the different grade of development of deciduous incisors and dental germs of permanent incisors can be identified radiographically. The purpose of the present study was to gain further insight into the radiographic appearance of deciduous incisors and dental germs of permanent incisors of horses in the period prior to and at the beginning of teeth shedding.

## 2. Materials and Methods

Twenty–five horse skulls were studied in the present study. Neither ponies nor draft horses were included in the study. Equine skulls, obtained from the Anatomy Department of the Veterinary School of Cordoba University, Spain, kept their complete dentition and surrounding bone without noticeable abnormalities. 

### 2.1. Visual Inspection

Based on published aging guidelines for horses [11,15,23], the whole skulls were visually examined by one of the authors, a specialist on equine dentistry (C.M., board-certified specialist, European Veterinary Dental College). Eruption of deciduous and permanent maxillary and mandibular incisors, morphological characteristics of their erupted crowns, and the presence of some of the permanent cheek teeth were the main criteria for determining the estimated age. Incisors were named according to the anatomical nomenclature, such as first, second, and third [25]. Correspondence with the modified Triadan system, worldwide used in veterinary dentistry [26], was made, appearing between en-dash after the corresponding anatomical name. Skulls with all deciduous incisors erupted and their occlusal surfaces in the same plane, assessed as between 12 and 30 months, were included in group 1. Skulls maintaining their second and third deciduous incisors—02 and 03; Quadrants 5,6,7, and 8—and with first permanent incisors—01; Quadrants 1,2,3, and 4—as well as second and maybe third permanent premolars—06 and 07; Quadrants 1,2,3, and 4—erupted, assessed as between 30 and 42 months of age, were included in group 2.

### 2.2. Radiographic Study

After visual inspection the maxillary incisor arcades were radiographed with an X-ray machine (Odel model C306-20^®^, Monza, Italy). Radiographs were processed by computerized radiology (Fuji Computed Radiography, Capsule XL, CR-IR 356^®^ Tokyo, Japan). Radiographs were obtained according to the standard procedures of intraoral dorsoventral technique, being the X-ray beam directed 90° to the plane that bisects the angle between the 1st maxillary incisors and the imaging plate [1,3,4,22,27]. Radiographs, being the incisors crown down and the horse’s left side presented to the viewer’s right, were then assessed [3]. Maxillary deciduous incisors were identified and the radiological characteristics of their erupted and nonerupted portions and surrounding bone were assessed. Dental germs of the maxillary permanent incisors were identified, and their radiological characteristics and surrounding bone were analyzed. In skulls in which maxillary permanent incisors were identified, their radiological characteristics of the erupted and non-erupted portions and surrounding incisive bone were assessed. A first analysis of the intraoral radiographs of group 1 skulls allowed us to identify the dental germs of the 1st maxillary permanent incisors—101 and 201—according to different grades of development. Some of the skulls—classified from now on as *group 1a*—revealed dental germs like round quite radiolucent areas and apparently no other notorious characteristics. Several skulls—organized from now on within *group 1b*—showed circumscribed radiopaque images within the respective radiolucent zones of the dental germs. In radiographic studies of some other skulls—from now on as *group 1c*—unerupted 1st permanent incisors—101 and 201—were identified as being short, conic, wide, and with hardly any observable radiopacity. A group of skulls—from now on *group 1d*—showed larger unerupted 1st permanent incisors—101 and 201—with greater radiopacity. Table 1 shows the distribution of the skulls in the above referred groups and some of the most remarkable radiographic characteristics of 1st permanent maxillary incisors—101 and 201—used to classify them. 

### 2.3. Computed Tomography Imaging

Subsequently, skulls were scanned with a helical CT scanner (CT Hi Speed CT/e Dual, General Electric Yokogawa Medical Systems LTD^®^, Hino, Japan) to obtain CT images from the incisors to the canines. Skulls were placed with the mandible on the CT scanning table. After image acquisition, CT slices from different planes of the incisors and adjacent structures were chosen, exported in DICOM format, and analyzed in Horos © software (open source for Apple, 64 bit, version 3.3.6). CT characteristics of deciduous incisors, dental germs of permanent incisors, permanent incisors (when they were present), and surrounding bone were analyzed. 

### 2.4. Length Measurements and Proportions

**Figure 1:**
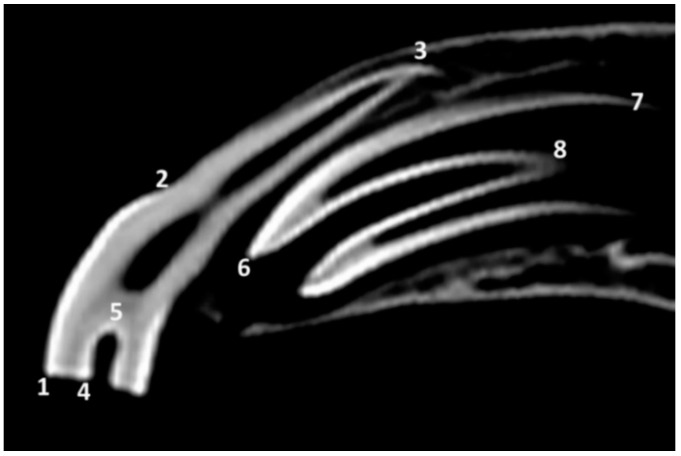
Sagittal computed tomography (CT) scan at midlevel of the 1st left maxillary deciduous incisor—601—of a skull of group 1d (supposedly the oldest among those with estimated age of between 12 and less than 30 months) showing the reference points for length measurements in the left maxillary 1st deciduous incisor—601— and in the unerupted 1st left permanent incisor—201. **1** and **6**, most occlusal points of the labial side of the teeth; **2** and **7**, most apical points of the labial enamel cover of the teeth; **3**, most apical point of the labial side of the 1st left maxillary deciduous incisor—60—; **4** and **6**, most occlusal points of the infundibular enamel, labial side; **5** and **8**, most apical points of the infundibular enamel, labial side.

Some length measurements and proportions were analyzed as a complementary study. Due to the small number of skulls per group, no statistical analysis was performed. Based on certain anatomical points (Figure 1), the following distances in mm were measured in sagittal scans at midlevel of the 1st deciduous incisors—501 and 601—by using Horos © software (open source for Apple 64 bit, version 3.3.6):

Length of the tooth (LTOOTH): distance between the most occlusal point and the most apical point of the labial side of the tooth.

Length of the crown (LCR): distance between the most occlusal point of the labial side and the most apical point of the labial enamel cover (i.e., dental crown) of the tooth.

Length of the infundibulum (LINF): distance between the most occlusal point and the most apical point of the infundibular enamel, both on labial side.

Based on the above measurements, the following percentage measurements were also calculated: 

Relative length of the crown (%CR): percent of length of the crown with respect to the total length of the same tooth.

Relative length of the infundibulum (%INF): percent of length of the infundibulum with respect to the length of the same tooth.

When the unerupted 1st first maxillary permanent incisors—101 and 201—were identified, the same measurements were taken. Until they erupt, the 1st permanent incisors—101 and 201—were considered as a whole tooth or crown. Once these teeth had erupted, the same measurements were taken on sagittal scans at midlevel of the tooth. Using the measurements for each group, the mean and standard deviations were calculated based upon the corresponding values of the right tooth and left tooth. 

## 3. Results

### 3.1. Visual Inspection

**Figure 2:**
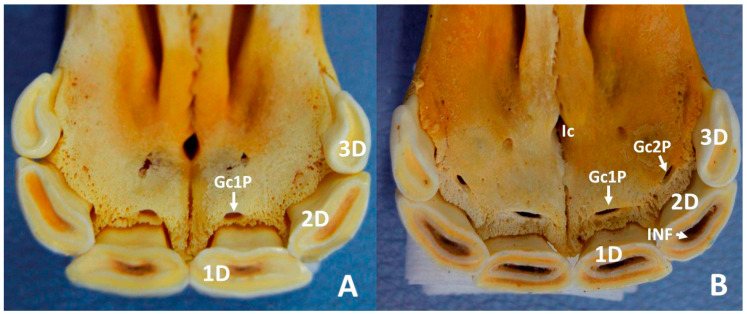
Ventral view of maxillary dental arcade, incisive part, of skull of group 1a (**A**) and of group 1d (**B**). **1D**, right 1st deciduous incisor—501; **2D**, right 2nd deciduous incisor—502; **3D**, right 3rd deciduous incisor—503; **Gc1P**, gubernacular canal of right 1st permanent incisor—101; **Gc2P**, gubernacular canal of right 2nd permanent incisor—102; **Ic**, incisive canal; **INF**, infundibulum of the right 2nd deciduous incisor—502.

Examination of the teeth of the skulls of group 1, classified as having an estimated dental age of between 12 and less than 30 months, revealed that all the deciduous incisors were clearly erupted and with wear on the occlusal surface of the 3rd incisors—03, Quadrants 5, 6, 7, and 8. They were of small size, with the oval occlusal surfaces in a mesiodistal orientation, and possessed shallow infundibula. Upon visual inspection, the most notable result available from the skull incisors of group 2, classified as having an estimated dental age of between 30 and less than 42 months, was the presence of the 1st permanent incisors—01, upon erupted Quadrants 1,2,3, and 4. In this group of skulls all other incisors were deciduous. In addition to the above, the skulls of group 2 had erupted the second permanent premolars or the second and third permanent premolars. In some of the skulls of group 2, there were the remains of the 1st deciduous incisors—01, Quadrants 5–7 and 8—in contact with the labial surface of the corresponding permanent tooth. Small oval openings, termed as gubernacular canals of the permanent incisors, were identified in the palatal region of the alveolar bone. They were present close to the 1st maxillary deciduous incisors—501 and 601—in groups 1a, 1b, and 1c (Figure 2A). In one of the skulls of group 1c the gubernacular canals were also present close to the 2nd deciduous incisors—502, 602. In group 1d the gubernacular canals were present close to the 1st and 2nd deciduous incisors—501, 502, 601, and 602—(Figure 2B). In group 2, there were gubernacular canals close to the 2nd and 3rd deciduous incisors—502, 503, 602, and 603—and in one of the skulls there was also a gubernacular canal close to the erupted 1st left permanent incisors—201.

### 3.2. Radiographic and CT Studies

**Figure 3:**
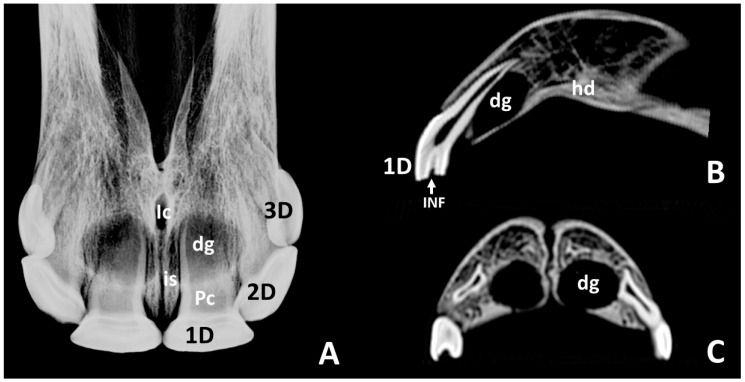
Intraoral radiographic image (**A**) and sagittal and transverse scans (**B**,**C**) of maxillary incisor arcade of a skull representative of group 1a. **1D**, left 1st deciduous incisor—601; **2D**, left 2nd deciduous incisor—602; **3D**, left 3rd deciduous incisor—603; **Pc**, pulp cavity; **dg**, dental germ of left 1st permanent incisor—201; **hd**, hard palate; **Ic**, incisive canal; **INF**, infundibulum of the left 1st deciduous incisor—601; **is**, interincisive suture.

In radiographic studies of skulls of *group 1a* (Figure 3A), the 1st deciduous incisors—501 and 601—were identified with an elongate shape. These teeth stick out approximately one third from the incisive bones. This part was widened in a mesiodistal direction. Approximately two thirds of these teeth were embedded in the incisive bone. The embedded parts of these teeth comprised a central elongated and radiolucent area with two collateral radiopaque fringes. They may be identified, respectively, as the pulp cavity (the cavity of the tooth that contains the pulp) and the surrounding hard component of the dental root. The apical portion of these teeth end at the level of the incisive canal. Lateral to the image of the 1st deciduous incisors—501 and 601—overlapping images of the second and third deciduous incisors—502, 503, 602, and 603. In the apical parts of the 1st deciduous incisors—501 and 601—rounded radiolucent zones identify the dental germs of the 1st permanent incisors—101 and 102. These zones almost medially reached the interincisive suture, and their caudal limits were just at the level of the incisive canal. Sagittal CT images at midplane of 1st deciduous incisors—501 and 601—(Figure 3B) showed these teeth convexly curved on the labial face and concavely curved on the lingual face. They tapered evenly from their occlusal part to their apex. Analysis of the structural density allowed us to identify in them the anatomical crowns (parts with peripheral enamel) and dental roots (parts without superficial enamel). The enamel cover (i.e., crown) extended more apically on the labial side than in the palatal side. Most parts of the crowns had erupted, and the all dental roots were enclosed within the alveolus. Scans showed shallow infundibula filled at their bottom with hyperdense tissue. Transversal and sagittal images (Figure 3B,C) showed round hypodense areas touching the palatal side of the root of the corresponding 1st deciduous incisors—501 and 601. These areas corresponded with the dental germs of the 1st permanent incisors—101 and 201. The dorsal tip of the dental germs reached the end of the apical height of the root of the 1st deciduous incisors—501 and 601—and it is limited caudally and ventrally by the more rostral portion of the hard palate. 

**Figure 4:**
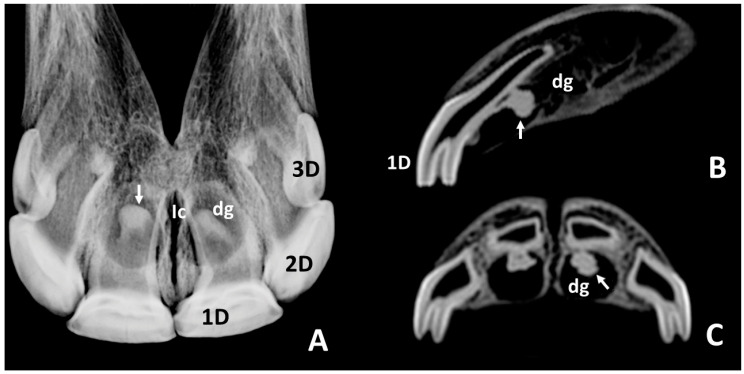
Intraoral radiographic images (**A**) and sagittal and transverse scans (**B**,**C**) of the maxillary incisor arcade of a skull representative of group 1b. **1D**, left 1st deciduous incisor—601; **2D**, left 2nd deciduous incisor—602; **3D**, left 3rd deciduous incisor—603; **dg**, dental germ of left 1st permanent incisor—201; **Ic**, incisive canal; **white arrows**, circumscribed radiopaque (**A**) and hyperdense (**B**,**C**) images within the dental germs of left 1st permanent incisor—201.

Intraoral radiographs of skulls of *group 1b* (Figure 4A) did not show notable differences in images of the 1st deciduous incisors with that of the same teeth in group in 1a. However, it was notable that the radiolucent zones of dental germs of the 1st permanent incisors—101 and 201—were in this group smaller, more elongated, and caudally went beyond the incisive canal. The most remarkable aspect of the radiographic study in this group was the presence of circumscribed radiopaque images within the respective radiolucent zones of the dental germs (white arrow, Figure 4A). CT scans of skulls in this group (Figure 4B,C) showed, as in the group 1a, most of the crown of the 1st deciduous incisors—501 and 601—already erupted, and their dental roots enclosed within the alveolus. As it was in the previous group, the enamel cover extended more apically in the labial side than in the palatal side. There were characteristic hypodense and oval areas of dental germs in permanent incisors—101 and 201—touching the palatal sides of the roots of the corresponding deciduous teeth. Within the dental germs circumscribed hyperdense images could be clearly distinguished (white arrows, Figure 4B,C). Sagittal scans showed that the dorsal limit of the dental germs did not dorsally reach the more apical point of the root of the corresponding deciduous tooth.

**Figure 5:**
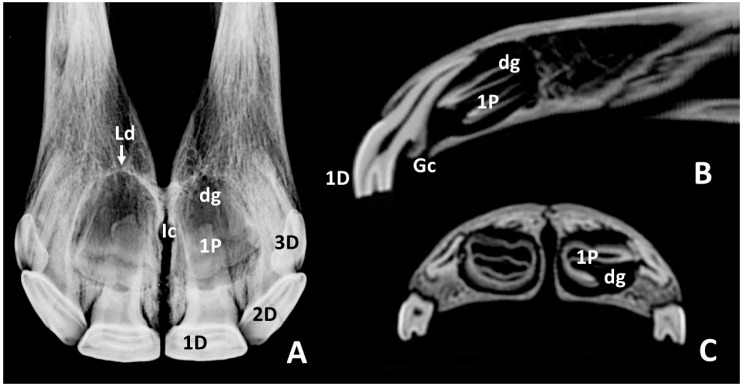
Intraoral radiographic images (**A**) and sagittal and transverse scans (**B**,**C**) of maxillary incisor arcade of one skull representative of group 1c. **1D**, left 1st deciduous incisor—601; **2D**, left 2nd deciduous incisor—602; **3D**, left 3rd deciduous incisor—603; **1P**, left unerupted 1st permanent incisor—201; **dg**, dental germ of left 1st permanent incisor—201; **Gc**, gubernacular canal of left 1st permanent incisor—201; **Ic**, incisive canal; **Ld**, lamina dura of the alveolus of the right 1st permanent incisor—101.

In intraoral radiographs of skulls of *group 1c* (Figure 5A), 1st deciduous incisors—501 and 601—showed a trapezoidal occlusal surface area. Wide radiolucent zones of dental germs of 1st permanent incisors—101 and 201—were visible in the body of every incisive bone. Their caudal limits exceeded the level of the incisive canal. Fine radiopaque lines enclosed the radiolucent areas of the germs in some of the skulls. They were considered the lamina dura of the alveolus. Wide and conic images of the unerupted crowns of 1st permanent incisors—101 and 201—could be identified within the radiolucent areas of the germs with hardly any observable radiopacity. Sagittal and transverse CT scans of skulls of this group (Figure 5B,C) showed the crown of the 1st deciduous incisors—501 and 601—to be erupted with the dental root within the alveolus. Images of the dental germs of the 1st permanent incisors—101 and 201—were touching the palatal sides of the roots of their corresponding deciduous teeth. In sagittal scans the dental germs of 1st permanent incisors—101 and 201—showed oval hypodense areas containing parallel hyperdense structures, which correspond to the unerupted crowns during early mineralization. The surrounding limits of the germs clearly exceeded dorsocaudally the apical point of the root of the corresponding deciduous tooth. It is noticeable that the ventral limits of the germs were disrupted caudally by a few millimeters to the corresponding deciduous incisors (Figure 5, Gc). This structure was identified as the gubernacular canal of the 1st permanent incisors—101 and 201. As stated above, within the hypodense area of the germs, the short crowns of the 1st permanent incisor—101 and 201—were identified. In them, the enamel did not reach the apical point of the infundibulum. 

**Figure 6:**
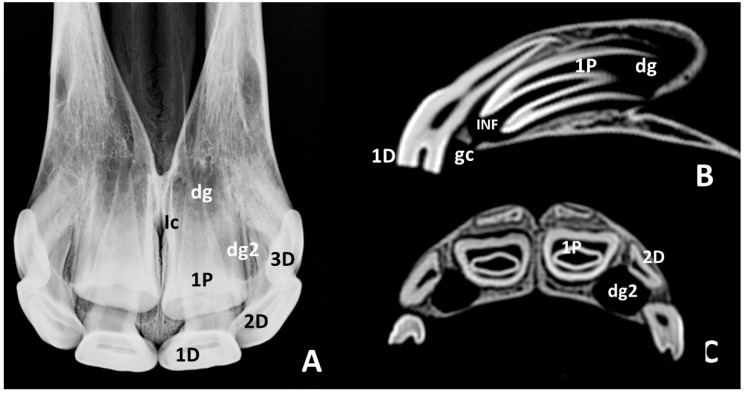
Intraoral radiographic images (**A**) and sagittal and transverse scans (**B**,**C**) of maxillary incisor arcade of one skull representative of group 1d. **1D**, left 1st deciduous incisor—601; **2D**, left 2nd deciduous incisor—602; **3D**, left 3rd deciduous incisor—603; **1P**, unerupted left 1st permanent incisor—201; **dg**, dental germ of left 1st permanent incisor—201; **dg2**, dental germ of left 2nd permanent incisor—202; **Gc,** gubernacular canal of left 1st permanent incisor—201; **Ic**, incisive canal; **INF**, infundibulum of the unerupted left 1st permanent incisor—201.

In intraoral radiographs of skulls of *group 1d* (Figure 6A), those images of the 1st deciduous incisors—501 and 601—had notably shorter lengths than those in all previously reported groups. The area corresponding to the occlusal surface was trapezoid. Relatively caudal to the 1st deciduous incisors—501 and 601—the crowns of unerupted 1st permanent incisors—101 and 201—were identified clearly (Figure 6, 1P). They were larger and of greater radiopacity than in the radiographs of skulls of group 1c. They tapered apically, where they were surrounded by radiolucent areas, smaller than in previous groups. Most parts of these radiolucent areas were caudal to the incisive canal. As it was also reported in some of the skulls of group 1c, a thin radiopaque line, the lamina dura, enclosed the radiolucent area of the dental germs in two of the three skulls of this group. The lamina dura was not identified in the radiograph shown in Figure 6. It was noticeable that lateral to the crowns of the unerupted 1st permanent incisors—101 and 201—two radiolucent images can be easily identified. They corresponded with the dental germs of the 2nd permanent incisors—102 and 202—(Figure 6, dg2). Sagittal CT scans at midplane of 1st deciduous incisors—501 and 601—(Figure 6A) showed the roots of these teeth hardly enclosed by osseous tissue. Hence, a great part of the roots were not included within the alveolus. As shown in previous groups, the enamel cover of the 1st deciduous incisors—501 and 601—i.e., crowns, extended more apically on the labial side than in the palatal side. Caudal to the root of the 1st deciduous incisors—501 and 601—the dental germs of the 1st permanent incisors—101 and 201—were shown, with large unerupted crowns occupying most of the space (Figure 6, 1P and dg). The length of the unerupted crowns of 1st permanent incisors—101 and 201—were apparently larger than in any of the previously reported groups. The infundibulum (Figure 6B, INF) of the unerupted incisors were quite large and completely formed by calcified tissue, from its occlusal part to the apical point. In sagittal scans at midlevel of the 2nd deciduous incisors—502 and 602—(not shown in the Figure 6) and in transverse scans (Figure 6C) two small hypodense areas were identified (Figure 6C, dg2) laterally to the dental germs of the 1st permanent incisors—101 and 201—and caudally to the roots of the 2nd deciduous incisors—502 and 602. They corresponded with the dental germs of the 2nd permanent incisors—102 and 202. 

**Figure 7:**
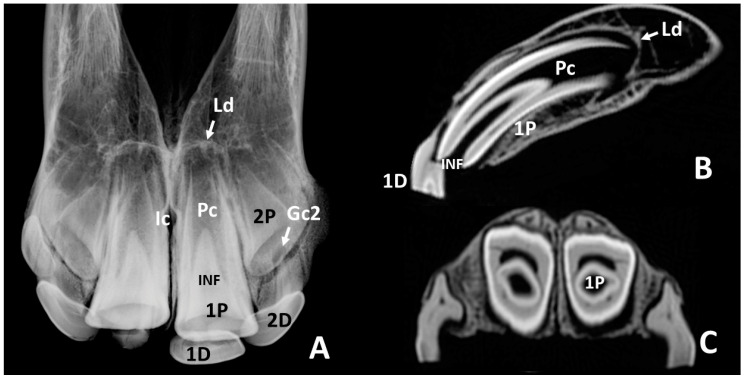
Intraoral radiographic images (**A**) and sagittal and transverse scans (**B**,**C**) of maxillary incisor arcade of one skull representative of group 2. **1D**, remain of left 1st deciduous incisor—601; **2D**, left 2nd deciduous incisor—602; **1P**, left 1st permanent incisor—201; **2P**, unerupted left 2nd permanent incisor—202; **Pc**, pulp cavity of the left 1st permanent incisor—201; **Gc2**, gubernacular canal of 2nd permanent incisor—202; **Ic**, incisive canal; **INF**, infundibulum of the left 1st permanent incisor—201; **Ld**, lamina dura of the alveolus of the left 1st permanent incisor—201. A small remain of right 1st deciduous incisor—501—is also present on the labial side of the corresponding permanent tooth.

In radiographs of skulls of *group 2* (Figure 7A) the 1st permanent incisors—101 and 201—appeared quite large. Most of the apical portion of these teeth was occupied by a wide radiolucent zone that ended rostrally in two horns. This zone corresponded with the pulp cavity (Figure 7, Pc). Collaterally they were limited by two radiopaque bands. Rostrally, a radiopaque triangular zone, between the horns of the pulp cavity, corresponded with the infundibulum, which in the more occlusal part of the tooth ended in an oval and slightly radiolucent image. In some of the radiographs of skulls of this group, remains of the 1st deciduous incisors—501 and 601—could be identified related with the labial side of the occlusal surface of the deciduous incisors. Laterally, with respect to the apical part of the 1st permanent incisors—101 and 201—shorter and conic crowns of the unerupted 2nd permanent incisors—102 and 202—were identified, in an oblique position. Surrounding the apex of the 1st and 2nd permanent incisors—101, 201, 102, and 202—there were semicircular radiopaque lines, which represented the lamina dura of the alveoli. In some skulls, small radiolucent areas could be identified at the level of the labial side of the occlusal surfaces of the unerupted 2nd permanent incisors—102 and 202. Sagittal scans at these levels confirmed that these images corresponded to the gubernacular canals of the 2nd permanent incisors—102 and 202. Sagittal CT scans at the level of the median plane of the 1st permanent incisors—101 and 201—(Figure 7B) showed most of these teeth enclosed by the corresponding alveoli on the dorsal and palatal sides, where a slight hyperdense line, the lamina dura, was clearly identified. A complete view of the internal morphology and structure of these teeth could be assessed. The hypodense pulp cavity occupied most of the apical part of the tooth, and it also extended within the occlusal part of the tooth, rostrally to the infundibulum. As stated for deciduous incisors, the enamel cover extended more apical in the labial side than in the palatal side of the permanent incisors. All the teeth, the surrounding soft tissues, and the lamina dura of the corresponding alveoli were clearly identified. The different grades of density allowed us to recognize of the structural components of the teeth. Hence, the 1st permanent incisors—101 and 201—of the skulls of this group showed the apical part of the infundibulum filled with cementum. The rostral surface of the incisive bone, covering the labial side of the incisors, was quite thin and remains of the 1st deciduous incisors—501 and 601—might still be attached to a part of the skulls. 

### 3.3. Length Measurements and Proportions

Due to the lack of statistical analysis, the results of the present section must be taken with caution. Results of length measurements for 1st deciduous incisors—501 and 601—and 1st permanent incisors—101 and 201—in sagittal scans at midlevel of the corresponding 1st incisors are shown in Table 2. Since in group 1c and 1d the enamel seemed to cover the whole surface of the unerupted 1st permanent incisors—101 and 201—the length of the tooth and length of the crown of these teeth were considered to be the same in these two groups.

The lengths of the crown and infundibulum of the 1st deciduous incisors—501 and 601—decreased from groups 1a and 1b to groups 1c and 1d. In the 1st permanent incisors—101 and 201—the lengths of the tooth, crown, and infundibulum increased from the unerupted teeth (groups 1c and 1d) to the erupted teeth (group 2). The lengths of the tooth, crown, and infundibulum of the 1st incisors were notably greater in permanent teeth—101 and 201—(i.e., group 2) than in deciduous teeth—501 and 601—(i.e., group 1).

Results of relative lengths of the crown and infundibulum as a percentage of the total length of the tooth for the 1st deciduous incisors—501 and 601—and 1st permanent incisors—101 and 201—are presented in Table 3. In groups 1c and 1d, the enamel seemed to cover the whole surface of the unerupted permanent teeth. Hence, the relative length of the crown (% CR) was considered 100% of the length of the tooth.

In the 1st deciduous incisors—501 and 601—the relative lengths of crown and infundibulum decreased from groups 1a and 1b to groups 1c and 1d. The relative lengths of the crown and infundibulum of the 1st incisors were notably greater in the permanent teeth (i.e., group 2) than in the deciduous teeth (i.e., groups 1).

## 4. Discussion

As stated by some authors [12,15,18] when determining a horse’s age by its incisors, the eruption dates and changes in appearance of the occlusal surfaces represent the main criteria. The chronology and sequence of eruption of the permanent cheek teeth are of great assistance for this purpose. Based on the published aging guidelines [11,15,17] and the expertise of one of the authors in equine dentistry, in the present study, the skulls were firstly classified as having between 12 and less than 30 months of age (group 1) or between 30 and less than 42 months of age (group 2). To avoid inaccuracies in teeth eruption times resulting from differences between breeds and types of horses, neither ponies nor breeds that differed from the general dental aging system have been employed in the study. Radiography has been widely used in horses for diagnoses and treatment purposes [2,4,21,22], and it can also provide valuable information from fossil sites about the life history, the age at death of the individuals and the evolution of past species [20]. In the present study, intraoral radiographs of the maxillary incisor arcades were used for selection purposes. In our opinion, the horses’ skulls in group 1 have a relatively wide range of ages and were divided into four different groups according to the development of their dental germs, as appeared in their respective radiographs. The complex and overlapping arrangement of the incisors within the maxillary arcade hampers the description of the three-dimensional positions of the individual teeth, especially while evaluating their 2D radiographs [4,14]. The CT scans obtained in the present study helped us better understand the internal structure of the erupted incisors, the dental germs of the unerupted incisors, and also the periodontal structures. They were also suitable to obtain some length measurements and proportions, which complemented the information supplied by the visual inspection and the radiographic study. In young horses before shedding, we can use radiographs to determine the degree of development of their corresponding deciduous incisors as well as the dental germs of their permanent incisors before shedding. Without adequate sedation, live horses would not tolerate the placement of the imaging plate in the mouth without chewing [3]. Hence, the authors of the present study are aware of the added requirements of repeating the study in live animals. 

### 4.1. Deciduous Incisors: Radiographic and CT Studies

The horses’ incisor teeth are connected together to form a continuous arch in each arcade and are thus implanted so that their roots converge [28]. Although all six maxillary incisors could be analyzed by intraoral radiographs, the 1st deciduous and permanent incisors—501,601,101, and 201—could be studied better, because they were positioned in the middle of the images and with less superimposition. Equine incisors feature a single Y-shaped pulp cavity made up of two pulp horns, labially to the infundibulum, and a single oval cavity more apically [14,21]. The referred configuration is present in both arcades but appears more pronounced in the maxillary incisors [14]. By means of intraoral radiographs, the referred Y-shaped configuration could be identified in the 1st permanent incisors—101 and 201—of group 2, and sagittal scans of these teeth showed the pulp horns to be located labially to the infundibulum. In the radiographs of all the skulls of group 1, the pulp cavity appeared as a radiolucent area located in the embedded parts of the teeth. Sagittal scans determined the extension and position of the occlusal and apical parts of the pulp cavity within the tooth. Sagittal scans were also of great use in assessing other aspects of the internal morphology and structure of the incisors. It has been studied that, in the horse’s incisors, the enamel cover, i.e., crown, extends more apically in the labial side than in the palatal side [14]. Sagittal CT scans obtained in the present study confirmed this non-uniform extension of the enamel cover in deciduous incisors. According to some authors, in horses, a large portion of the dental crown (the enamel-containing part of the tooth) of incisors lies within the dental alveolus [10,13,29]. Although not mentioned in the latest articles, it is supposed that the above statement was made in reference to permanent incisors. The same was found for the 1st permanent incisors—101 and 201—of the skulls of group 2 of the present study, since most of them were enclosed by their corresponding alveoli. It has been described that, after eruption, the large crowns of the horse’s teeth are only gradually extruded, and the delayed development of the roots allows growth to continue for some years after the teeth have come into wear [10,13,28]. The unerupted crown of an equine’s teeth functions as a root to counterbalance their abrasive diet and to support the strong stresses generated during mastication [29]. In the present study, it was observed that the majority of the crown of the 1st deciduous incisors—501 and 601—of skulls of groups 1a to 1c were erupted, contrary to what was observed for permanent incisors—101 and 201. In the groups 1a to 1c, the position of the crowns with respect to the alveoli was more similar to that of brachyodont teeth, such as primates or dogs, which have the entire crown exposed in the oral cavity [10,28,29]. In skulls of group 1d, supposedly the oldest of estimated ages between 12 and less than 30 months, a great part of the root of the deciduous incisors were not embedded within the alveolus, probably due to the impending dentition change. 

### 4.2. Permanent Incisors and Alveoli: Radiographic and CT Studies

Intraoral radiographs and CT scans were used in the present study to analyze the permanent incisors, from the state of the dental germ to the erupted teeth. Odontogenesis in the horse follows the same principles and involves the same sequential processes as seen in other mammals [10]. The first period comprises the formation of the gingival epithelium of the fetal oral cavity of the so-called “dental lamina” and, later, its subsequent separation into cyst-like structures known as “tooth buds” [10,11]. Although both the deciduous and permanent tooth buds form within a short time of each other, the tooth buds for permanent teeth remain dormant until the mandible and maxilla obtain the sufficient length to provide space for their development [11]. In the second period, cellular differentiation and interaction with the surrounding mesenchyma leads to the formation the tooth germs [10]. After that, a complicated process of growth and remodeling of the tooth germs, and production of the tooth substances leads to the fully developed teeth [10,13], deep into the roots of the temporary set of equivalent teeth [28]. After completing the development of the permanent incisors, the deciduous incisors are displaced labially by the erupting permanent incisors [10], which usually erupt on the lingual aspect of the corresponding deciduous teeth [18]. The latest process involves continuous adjustment and remodeling of the alveoli and surrounding bone [28]. Intraoral radiographs of skulls obtained in the present study were suitable to identify the dental germs of the 1st permanent incisors—101 and 201—and 2nd the permanent incisors—102 and 202—when they were present. CT scans confirmed the position and morphology of these dental germs. Some authors have shown in equines that tooth germs of some permanent molar teeth that do not have predecessor deciduous teeth (4th cheek teeth) appear radiographically at 6 to 7 weeks of age and early crown mineralization of them is detected at 7 weeks of age [19]. The same authors have showed radiographic evidence of tooth germs in premolar teeth that have predecessor deciduous teeth (1st to 3rd cheek teeth) at 10 to 12 months of age and early signs of mineralization at 11 to 13 months of age. The dental germs of the 1st permanent incisors—101 and 201—in the present study were identified radiographically in the skulls of the horses of groups 1a (supposedly the youngest of the study), and the first sign of crown mineralization appeared in group 1b, supposedly being a few months older than the previous ones. In skulls of the horses of groups 1c and 1d (supposedly older than horses of groups 1a and 1b, but also with an estimated age of between 12 and less than 30 months), the crowns of the 1st permanent incisors—101 and 201—were mineralized. CT scans of these last skulls (groups 1c and 1d) showed the crowns of the 1st permanent incisors—101 and 201—are clearly hyperdense due to their grade of calcification. Intraoral radiographs of a yearling (between one and two years old) showed the dental germs of the 1st permanent incisors—101 and 201—to have a similar grade of development and mineralization as the skulls of group 1c of the present study [22]. All the above may indicate that the dental germs of the 1st permanent incisors—101 and 201—may appear radiographically shortly before 12 months of age, and that the first sign of mineralization would appear some months later.

It has been described that at the periphery of the dental germ, cell differentiation, and calcification organize the individual constituents of the periodontium, i.e., the cementum, the periodontal ligament, and the alveolar bone [13]. Periodontal research has demonstrated that the periodontium, in addition to fixing the tooth within the alveolus, it also possesses a shock-absorbing and reparatory function and also contains multiple elements, such as mesenchymal cells, capable of transforming into any of the periodontal tissues when repair is needed [9]. Alveolar bone is very flexible and constantly remodels to accommodate the changing shape and size of the dental structures it contains [10,18]. The intraosseous stage of tooth eruption needs resorption of bone in the direction of eruption and formation of bone on the opposite side [10]. These activities depend upon the adjacent parts of the true dental germ [30]. Sagittal CT scans at midplane of the 1st deciduous incisors—501 and 601—of the skulls of group 1d (Figure 6A) showed a substantial part of the root was not included within the corresponding alveoli. On the contrary, CT scans of the 1st permanent incisors—101 and 201—of group 2 (Figure 7A) showed these teeth enclosed by the corresponding alveoli on the dorsal and palatal sides. The above may demonstrate in equines that bone resorption and bone formation are also polarized around erupting incisor teeth. The inner wall of the alveolar bone, a thin layer of compact bone, has been shown to appear radiographically as a delicate radiopaque line called the lamina dura [10,11,13,18]. It has been stated that the lamina dura is not detectable on computed tomography [11]. We disagree with this statement, because in the present study, the lamina dura of the alveoli of the 1st permanent incisors—101 and 201—was detected radiographically in some of the skulls of groups 1c and 1d and in all the skulls of group 2. Confirmation of the presence of this osseous part of the alveoli was confirmed by the hyperdensity shown in the structure in sagittal CT scans. 

By visual inspection of skulls of the present study, small oval openings were detected in the palatal region of the alveolar bone, close to the deciduous incisors. They were identified as the openings of the so-called gubernacular canals. In humans, the gubernacular canals have been defined as small channels that connect the bony crypt of the permanent unerupted teeth to the oral surface of the alveoli [30,31]. In the skulls of horses in the present study, they seemed to appear gradually from medial to lateral from group 1a to group 2, related to the grade of development and calcification of the corresponding dental germs found in radiographs. In one of the skulls of group 2, there was also a gubernacular canal close to the corresponding already erupted 1st permanent incisors—101 and 201. The gubernacular canal contains the gubernacular cord, a structure composed of conjunctive tissue, which links the tooth follicle (part of the dental germ) to the overlying gingiva [31]. Both the gubernacular canal and the cord were supposed to help guiding and directing the course of tooth eruption [31]. However, the existence and functions of the gubernacular canal and the cord in humans are still controversial and questioned [31]. To the authors’ knowledge there are no references in the literature describing the presence and the function of the gubernacular canal in horses. Therefore, studies on this issue are needed to deepen the knowledge of the role of the gubernacular canal and the cord in the eruption of the incisors in equines. Anyway, this structure is present in the change of dentition of equines, and it may appear on radiographs (and CT scans) of the incisor region, as was the case for group 2 (Figure 7, Gc2). Differential diagnosis using a possible pathological image must be employed in the corresponding cases.

### 4.3. Deciduous and Permanent Incisors: Lengths and Proportions

To the authors’ knowledge, there is no available data concerning tooth length of equine deciduous incisors. Hence, the length measurements and proportions obtained in the present study may complement the morphological data studied by radiographs and CT scans. However, due to the lack of statistical analysis, these results should be considered with caution. It has been studied that formation of permanent equine teeth is not finished once the teeth erupt [13]. Abrasion and mastication wear down the functional crown at a rate of 2 to 3 mm per year, but the reserve crown erupts continuously so that approximately 2 cm of exposed crown is maintained [11]. In the 1st deciduous incisors—501 and 601—of the present study, the relative lengths of the crown and the infundibulum decreased from groups 1a and 1b to groups 1c and 1d (i.e., the relative length of the root increased in the same manner), coincident with an increase of the total length of the tooth. These results may suggest the leading role of root elongation to outbalance the grade of occlusal wearing in deciduous incisors, as it had already been suggested for permanent incisors from two to four years post eruption [14]. The infundibulum of the equine incisors is an enamel infolding inside the occlusal surface [15] that, in horses, has a depth of 10–30 mm [10]. In the present study, the length of the infundibulum ranged between 4.5 ± 1.4 mm of group 1d and 35.9 ± 3.2 mm of group 2. Our results agree with what has been stated by some authors who affirmed that deciduous incisors contain shallower infundibula than their permanent successors [10]. Once formed, the length of the infundibulum cannot be shortened, except by being worn from the occlusal side [14]. Hence, it seems logical that, in the present study, the minimum length of the infundibulum of the deciduous incisors was found to exist in the supposedly older animals (group 1d). It has been affirmed that during eruption, hypsodont teeth have no true roots, i.e., with an apical enamel-free area [10]. We suppose that the above statement referred to permanent incisors, since in the present study the length of the root in the erupted 1st permanent incisors—101 and 201—(group 2) represented less than 13 % of the tooth length (relative length of the crown 87.4 ± 5.6 mm). However, the length of the root in the 1st deciduous incisors—501 and 601—of the supposedly youngest animals (group 1a) represented more than 50% of the length of the tooth (relative length of the crown 48.5 ± 4.1 mm). 

From this study, veterinary clinicians might improve their knowledge of the radiographic appearance of deciduous incisors and dental germs in the permanent incisors of horses in the period prior to teeth shedding. These radiographic data may be considered when injuries or disorders affect the maxillary incisor arcade. The main limitations of the present study were the lack of knowledge of the precise age of the horses to which the skulls used to belong and the small number of skulls in each group to perform a statistical analysis of the dental measurements and proportions. Further radiographic and CT investigations on a representative number of horses of known ages would provide a more comprehensive understanding of the development of dental germs of permanent incisors.

## 5. Conclusions

Radiographic intraoral images are suitable to identify the grade of development of the dental germs of the maxillary permanent incisors. In horses conforming to the general dental aging system, the dental germs of 1st permanent incisors—101 and 201—(identified radiographically as rounded radiolucent areas located at the level of the apical parts of the 1st deciduous incisors—501 and 601—appear at the age of twelve months. The first sign of crown mineralization of these dental germs appears a few months later. Before the teeth shedding, the unerupted mineralized crowns of the 1st permanent incisor can be identified radiographically as being relatively caudal to the corresponding 1st deciduous incisors. 

## Figures and Tables

**Table 1:** In 25 skulls of Spanish horses, most remarkable radiographic characteristics of 1st permanent maxillary incisors—101, 201—and number of skulls studied. Groups 1a to 1d, estimated age of between 12 and less than 30 months. Group 2, estimated age between 30 and 42 months.

Groups	Radiographic Appearance of 1st Maxillary Permanent Incisors—101, 201	Number of Skulls
1 1a	Dental germs, round radiolucent areas in the incisive bone No other notorious characteristics	5
1b	Dental germs, round radiolucent areas in the incisive bone Circumscribed radiopaque images inside	4
1c	Dental germs, unerupted crowns within the incisive bone, hardly any observable radiopacity	8
1d	Dental germs, larger unerupted crowns within the incisive bone, greater radiopacity	4
2	Erupted, quite large, and radiopaque	4

**Table 2:** Results (mean ± standard deviation, mm) of lengths of the tooth, crown, and infundibulum (LTOOTH, LCR, and LINF, respectively) of 1st deciduous—501 and 601—and permanent—101 and 201—incisors of horses with an estimated age of between 12 and less than 30 months (groups 1a–1d) and of between 30 and less than 42 months (group 2). A study of 25 horse skulls. Since in group 1c and 1d the enamel seemed to cover the whole surface of the unerupted 1st permanent incisor, length of the tooth and length of the crown of these teeth were considered to be the same in both of these two groups.

Group	1st Deciduous Incisors—501 and 601			1st Permanent Incisors—101 and 201		
	LTOOTH	LCR	LINF	LTOOTH	LCR	LINF
1a	39.4 ± 0.8	19.1 ± 1.8	7.3 ± 1.8			
1b	38.7 ± 1.5	18.9 ± 1.3	8.5 ± 1.8			
1c	41.4 ± 1.9	17.8 ± 1.9	7.3 ± 0.8	19.1 ± 2.9	19.1 ± 2.9	17.4 ± 3.2
1d	41.6 ± 1.8	15.1 ± 2.5	4.5 ± 1.4	35.1 ± 2.3	35.1 ± 2.3	31.2 ± 2.8
2				57.3 ± 3.8	50.2 ± 7.2	35.9 ± 3.2

LTOOTH: Length of the tooth, LCR: Length of the crown, LINF: Length of the infundibulum.

**Table 3:** Results (mean ± standard deviation) of relative lengths of the crown and infundibulum (% CR and % INF, respectively) of 1st deciduous incisors—501 and 601—and/or 1st permanent incisors—101 and 201—with respect to the total length of the tooth. Study of 25 horse skulls.

Group	1st Deciduous Incisors —501 and 601		1st Permanent Incisors —101 and 201	
	% CR	% INF	% CR	% INF
1a	48.5 ± 4.1	18.4 ± 4.3		
1b	48.8 ± 2.8	22.1 ± 4.5		
1c	43.1 ± 3.1	17.5 ± 2.3	100.0 ± 0.0	96.7 ± 7.2
1d	36.3 ± 6.5	10.9 ± 3.2	100.0 ± 0.0	88.9 ± 3.5
2			87.4 ± 5.6	62.8 ± 6.1

Relative length of the crown (%CR): percent of length of the crown with respect to the total length of the same tooth.Relative length of the infundibulum (%INF): percent of length of the infundibulum with respect to the length of the same tooth.
